# Absent fourth heart sound as a marker of adverse events in hypertrophic cardiomyopathy with sinus rhythm

**DOI:** 10.1111/anec.12932

**Published:** 2022-02-11

**Authors:** Chieko Sakai, Tatsuya Kawasaki, Hirofumi Kawamata, Kuniyasu Harimoto, Hirokazu Shiraishi, Satoaki Matoba

**Affiliations:** ^1^ 38379 Department of Cardiology Matsushita Memorial Hospital Osaka Japan; ^2^ 12898 Department of Cardiovascular Medicine Graduate School of Medical Science Kyoto Prefectural University of Medicine Kyoto Japan

**Keywords:** Exercise tolerance, fourth sound, hypertrophic cardiomyopathy, phonocardiography, prognosis

## Abstract

**Background:**

Patients with hypertrophic cardiomyopathy (HCM) in sinus rhythm commonly show the fourth heart sound (S4). The lack of S4 may be a marker of impaired atrial function in HCM patients with sinus rhythm.

**Methods and Results:**

This retrospective study consisted of 47 patients with HCM who had undergone phonocardiography and a cardiopulmonary exercise test. The primary outcome was a composite of cardiac death, stroke, hospitalization for worsening heart failure, and newly developed atrial fibrillation (AF). S4 was detected in 38 of 43 patients with sinus rhythm (88%). Peak oxygen consumption was the highest in 38 sinus rhythm patients with S4 (23.6 ± 5.6 mL/kg/min), middle in five sinus rhythm patients without S4 (19.3 ± 6.7 mL/kg/min), and lowest in four patients with AF (15.7 ± 3.3 mL/kg/min, *p* = 0.01). After a median of 40.5 months, the incidence of the primary outcome was higher in patients without S4 than in those with S4 (33% vs. 8%; hazard ratio, 6.17; 95% confidence interval, 1.02 – 37.4; *p* = .04) and higher in sinus rhythm patients without S4 than in those with S4 (60% vs. 8%; hazard ratio, 12.05; 95% confidence interval, 2.31 – 71.41; *p* = .007).

**Conclusions:**

The absence of S4 on phonocardiography was associated with impaired exercise tolerance and adverse cardiac events in HCM patients with sinus rhythm.

## 1 INTRODUCTION

Patients with hypertrophic cardiomyopathy (HCM) in sinus rhythm commonly show the fourth heart sound (S4) (Kawai et al., 1983; Sato et al., 2018). Since the cause of S4 is thought to be due to a forceful left atrial contraction into a non‐compliant left ventricle (Goodwin, 1982; Shaver et al., 1985), S4 may no longer be present not only in patients with atrial fibrillation (AF), but also in sinus rhythm patients with impaired atrial function (Abe et al., 1988; Vancheri & Gibson, 1989). Given that patients with HCM more frequently develop AF than the general population and that the presence of AF is associated with a poor prognosis (Guttmann et al., 2017; Kirchhof et al., 2016; Lee et al., 2017; Olivotto et al., 2001), sinus rhythm patients without S4 may be at risk of developing AF and adverse cardiac events in this disease entity. The objectives of this study were to examine the incidence of absent S4 in HCM patients with sinus rhythm and clarify its clinical significance.

## 2 METHODS

### 2.1 Study population

This retrospective study consisted of 47 consecutive patients with HCM (37 men; mean age, 64 years) who had undergone phonocardiography, a cardiopulmonary exercise test (CPET), and biomarker measurements at Matsushita Memorial Hospital. The diagnosis of HCM was based on the echocardiographic demonstration of left ventricular end‐diastolic thickness ≥15 mm in the absence of any cardiac or systemic disorder that may cause hypertrophy, such as severe hypertension defined as blood pressure ≥160/100 mmHg or aortic stenosis defined as an aortic valve area <1.5 cm^2^. Exclusion criteria were coronary heart disease, moderate or severe valvular heart disease, renal dysfunction defined as an estimated glomerular filtration rate <60 mL/min/1.73 m^2^, permanent mechanical device implantation, catheter ablation, septal myectomy, alcohol septal ablation, or heart transplantation.

All patients with HCM were in New York Heart Association class I or II. Among all patients examined, seven had taken beta‐blockers and none had taken amiodarone. Thirty patients had non‐obstructive asymmetric septal hypertrophy, seven had left ventricular outflow tract obstruction, seven had apical hypertrophy, and three had midventricular obstruction. Paroxysmal AF was defined as one or more electrocardiographically documented episodes of AF and sinus rhythm at the time of CPETs. Persistent AF was considered to be present when electrocardiographically documented AF was identified for more than seven days and was not actively being managed by a rhythm control strategy. Persistent AF was observed in four patients and none was diagnosed with paroxysmal AF. This study was approved by the Ethics Committee of Matsushita Memorial Hospital and informed consent for the assessment was obtained from all patients with HCM.

## 3 PHONOCARDIOGRAPHY

**FIGURE 1:**
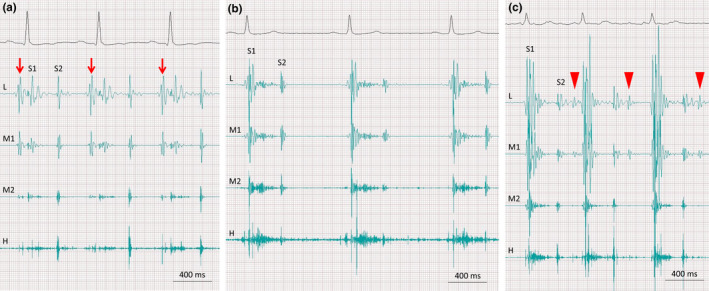
Representative results of phonocardiography. Case 1: A 74‐year old man with HCM and non‐obstructive asymmetric septal hypertrophy. The phonocardiogram shows the fourth heart sound (panel A, arrows) before the first heart sound (S1). Case 2: A 70‐year old man with HCM and non‐obstructive asymmetric septal hypertrophy. No extra heart sound was detected on the initial phonocardiography (Panel B), but AF developed 17 months later. Phonocardiography shows the third heart sound (Panel C, arrowheads) after the second heart sound (S2). All three phonocardiograms were recorded at the apex of the left ventricle in the semi‐left decubitus position on the same scale. H = high frequency; L = low frequency: M1 = lower‐middle frequency: M2 = higher middle frequency.

A phonocardiogram was obtained at the apex as well as at the right and left sternal borders in the half left lateral decubitus position or supine position at a paper speed of 50 or 100 mm/s using a commercially available device (MES‐1000, Fukuda‐Denshi Co., Tokyo, Japan) (Honda et al., 2016; Sato et al., 2018), as shown in Figure 1. Measurements included four frequencies: a low frequency, lower‐middle frequency, higher‐middle frequency, and high frequency. The cut‐off, attenuation, nyquist, and differential sensitivity were 50 Hz, −6 dB/Oct, 1, and −32 dB, respectively, for the low frequency; 50 Hz, −18 dB/Oct, 3, and −32 dB for the lower‐middle frequency; 160 Hz, −24 dB/Oct, 4, −16 dB for the higher‐middle frequency; and 315 Hz, −24 dB/Oct, 4, and 0 dB for the high frequency. When an apexcardiogram was recorded, a simultaneous apical phonocardiogram was obtained near the apex because the microphone of the apexcardiogram was placed exactly at the apex. S4 was considered to be present when a low frequency sound was recorded best at the apex between the onsets of the P wave and QRS complex of an electrocardiogram, coinciding with a large peaked A wave in an apexcardiogram if available (Tavel, 1971). S4 needed an amplitude of 1.0 mm or greater on the phonocardiogram with the above‐described settings (Sato et al., 2018). The presence or absence of S4 was assessed by two experienced cardiologists; disagreement was resolved by consensus.

### 3.1 CPET

All patients underwent symptom‐limited CPETs using an electronically braked cycle ergometer (BK‐ERG‐121, Fukuda‐Denshi Co., Ltd., Japan) and a breath‐by‐breath gas exchange measurement system (AE310S, Minato Medical Science Co., Ltd., Japan) without the discontinuation of medication prior to testing. A 12‐lead electrocardiogram and blood pressure were recorded at an interval of one minute during CPET using a stress system (ML‐6500 or ML‐9000 system, Fukuda Denshi Co., Ltd., Japan). Exercise was preceded by 3 min of resting breath‐by‐breath gas exchange monitoring, followed by a 3‐min unloaded warm‐up with 10 watts that was increased using a ramp protocol starting at 10 watts with a gradual increase of 10 watts every minute until patients stated that they had reached maximal effort. The rating of perceived exertion or the Borg scale was measured at the maximal effort (Borg, 1982).

All CPETs were analyzed by a single expert physician to measure oxygen consumption (VO_2_), VO_2_/workload relationship, anaerobic threshold (AT), and the ventilation versus carbon dioxide relationship during exercise (VE/VCO_2_ slope) in a standard manner (Magrì et al., 2016). In brief, predicted VO_2_ was assessed using the gender‐adjusted, age‐adjusted, and weight‐adjusted formula; AT was measured by a V‐slope analysis of VO_2_ and VCO_2_, and confirmed by ventilator equivalents and end‐tidal pressures of CO_2_ and O_2_; the VE/VCO_2_ slope was calculated as the slope of the linear relationship between VE and VCO_2_ from the first minute after the beginning of the loaded exercise and the end of the isocapnic buffering period. An abnormal blood pressure response was defined as a change in systolic blood pressure during the whole exercise period of <25 mm Hg from resting values.

## 4 BIOMARKERS

Serum levels of high‐sensitivity cardiac troponin T as well as plasma levels of atrial natriuretic peptide and brain natriuretic peptide were measured before CPETs using commercially available kits: Elecsys Troponin T hs (Roche Diagnostics GmbH, Mannheim, Germany) for high‐sensitivity cTnT; MI02 Shionogi ANP (Shionogi & Co., Ltd., Osaka, Japan) for atrial peptide; and MI02 Shionogi BNP (Shionogi & Co., Ltd., Osaka, Japan) for BNP, as previously described (Kawasaki et al., 2015).

## 5 ECHOCARDIOGRAPHY

An echocardiographic examination was performed by echocardiography equipment (Vivid E9; GE Healthcare, Milwaukee, WI) using a standard method (Lang et al., 2005). The left ventricular end‐diastolic diameter, left ventricular ejection fraction, left atrial end‐systolic diameter, mitral valve E wave peak velocity, and septal mitral annular early peak velocity (E’) were measured. Left ventricular outflow tract obstruction was considered to be present when the peak flow velocity was >2.5 m/s by the Doppler method under resting conditions or the Valsalva maneuver. Maximum left ventricular wall thickness was assessed not only on echocardiography, but also on cardiac magnetic resonance, if available, as previously prescribed (Sato et al., 2018).

## 6 FOLLOW‐UP AND ENDPOINT

All patients were followed up from CPETs. Patient information was obtained from available medical records and interviews with patients and/or their physicians in charge. The primary outcome was a composite of cardiac death, stroke, hospitalization for worsening heart failure, and the development of AF when sinus rhythm at the time of CPETs.

### 6.1 Statistical analysis

Categorical variables were compared by the chi‐squared test or Fisher's exact test as appropriate. Continuous variables were expressed as the mean ± standard deviation and compared between two groups using the Student's *t*‐test and between three groups by a one‐way analysis of variance. Variables regarding biomarkers were expressed as the median ± standard error and compared using the Mann–Whitney U test because of possibly skewed distributions. Time‐to‐event data were evaluated using Kaplan–Meier estimates and Cox’s proportional‐hazards models, stratified according to S4 on phonocardiography. Cox models were used to calculate hazard ratios, 95% confidence intervals (CI), and p‐values. A two‐sided *p* < .05 was considered to be significant.

## 7 RESULTS

S4 as assessed by phonocardiography was detected in 38 out of 47 patients (81%) and out of 43 patients with sinus rhythm (88%). No morphological differences were observed between patients without S4 and those with S4 (*p* = .85; e.g., non‐obstructive asymmetric septal hypertrophy in five and 25, and apical hypertrophy in one and six, respectively). Patient characteristics at baseline are shown in Table 1. The absence of S4 was significantly associated with a symptomatic status, persistent AF, larger left atrial dimension, and higher mitral valve E wave peak velocity. Left atrial dimensions were 40.0 ± 7.0 mm in sinus rhythm patients without S4 and 48.0 ± 4.6 mm in patients with AF; the mitral valve E wave peak velocities were 79 ± 19 and 75 ± 17 cm/s, respectively. In the biomarker analysis, patients without S4 had higher atrial natriuretic peptide and brain natriuretic peptide levels than those with S4. Atrial natriuretic peptide levels were 75.3 ± 50.0 pg/mL in sinus rhythm patients without S4 and 138.0 ± 16.6 pg/mL in patients with AF; brain natriuretic peptide levels were 85.4 ± 85.5 and 331.9 ± 49.6 pg/mL, respectively. An additional analysis after the exclusion of seven patients receiving beta‐blockers did not significantly alter the results obtained on patient characteristics at baseline (data not shown).

**TABLE 1:** Patient characteristics at baseline

	S4		p
	Absent (n = 9)	Present (n = 38)	
Age, years	67 ± 8	63 ± 11	.24
Male	6 (67%)	33 (87%)	.97
Height (cm)	168 ± 11	166 ± 7	.54
Body weight (kg)	67 ± 9	66 ± 10	.69
Body mass index (kg/m^2^)	24 ± 4	24 ± 3	.98
Family history of HCM	5 (56%)	13 (34%)	.21
Family history of premature death*	2 (22%)	4 (11%)	.32
Unexplained syncope	0	2 (5%)	.65
Asymptomatic status	3 (33%)	28 (74%)	.03
Persistent AF	4 (44%)	0	<.01
Echocardiography			
Left ventricular end‐diastolic diameter (mm)	44 ± 6	43 ± 5	.62
Left ventricular end‐systolic diameter (mm)	28 ± 5	27 ± 4	.38
Left ventricular ejection fraction (%)	64 ± 6	67 ± 6	.24
Interventricular septum thickness (mm)	12 ± 4	14 ± 5	.38
Left ventricular posterior wall thickness (mm)	10 ± 1	10 ± 2	.68
Maximum left ventricular wall thickness (mm)	18 ± 2	21 ± 7	.07
Left atrial dimension (mm)	43 ± 7	38 ± 5	.04
E (cm/s)	77 ± 17	60 ± 14	.02
Mitral annular E′ (cm/s)	5.3 ± 1.8	4.7 ± 1.5	.36
E/E′	15.8 ± 3.1	14.0 ± 4.9	.18
Left ventricular outflow tract obstruction	2 (22%)	5 (13%)	.40
Biomarker			
High‐sensitivity cardiac troponin T (ng/mL)	0.024 ± 0.011	0.012 ± 0.002	.13
Atrial natriuretic peptide (pg/mL)	118.0 ± 27.9	63.2 ± 9.3	.04
Brain natriuretic peptide, pg/mL	185.0 ± 58.2	55.7 ± 16.2	.02

Note

Data are means ± standard deviations or numbers (%). Biomarkers are means ± standard errors. *Death in first‐ or second‐degree relatives. HCM = hypertrophic cardiomyopathy; S4 = fourth heart sound.

**FIGURE 2:**
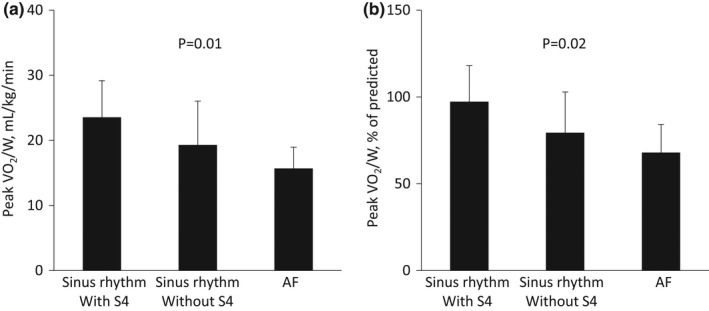
Exercise tolerance. Peak oxygen consumption (VO_2_) and its predicted values are the highest in sinus rhythm patients with fourth heart sounds (S4), followed by sinus rhythm patients without S4 and patients with AF.

Heart rates and systolic blood pressures at rest did not significantly differ between patients with S4 and those without S4, whereas heart rates at peak exercise were lower in patients without S4 than in those with S4 (Table 2). Although no significant differences were observed in the peak respiratory rate, peak respiratory exchange ratio, or the Borg score between the two groups, the absence of S4 was significantly associated with a reduced exercise capacity, such as lower peak metabolic equivalents, a lower peak VO_2_/W, and higher VE/VCO_2_ slope. An additional analysis after the exclusion of seven patients receiving beta‐blockers did not significantly alter the results obtained on CPETs (data not shown). Peak VO_2_/W and its predicted values were the highest in 38 sinus rhythm patients with S4 (23.6 ± 5.6 mL/kg/min and 97% ± 21%), middle in five sinus rhythm patients without S4 (19.3 ± 6.3 mL/kg/min and 79% ± 23%), and the lowest in 4 patients with AF (15.7 ± 3.2 mL/kg/min and 16% ± 2%), as shown in Figure 2.

**TABLE 2:** Results of CPETs according to S4

	S4		p
	Absent (n = 9)	Present (n = 38)	
Heart rate			
Rest (bpm)	69 ± 15	70 ± 14	.85
Peak (bpm)	126 ± 25	146 ± 21	.04
Systolic blood pressure			
Rest (mmHg)	136 ± 22	143 ± 25	.47
Peak (mmHg)	159 ± 39	185 ± 44	.10
Abnormal blood pressure response	4 (44%)	12 (32%)	.36
Peak respiratory rate (bpm)	35 ± 7	36 ± 9	.52
Peak respiratory exchange ratio	1.07 ± 0.09	1.12 ± 0.08	.21
Borg score	15.7 ± 2.6	15.4 ± 2.0	.82
Peak metabolic equivalents	5.1 ± 1.6	6.8 ± 1.7	.01
Load, w	89 ± 30	114 ± 34	.04
AT VO_2_/W (mL/kg/min)	10.3 ± 1.4	12.9 ± 2.5	<.01
AT VO_2_/W, % of predicted	63 ± 8	80 ± 16	<.01
Peak VO_2_/W (mL/kg/min)	17.9 ± 5.5	23.6 ± 5.6	.01
Peak VO_2_/W, % of predicted	74 ± 20	98 ± 21	.01
VE/VCO_2_ slope	32.4 ± 3.5	28.1 ± 7.7	.02

Note

Data are means ± standard deviations or numbers (%). CPET = cardiopulmonary exercise test; S4 = fourth heart sound.

**FIGURE 3:**
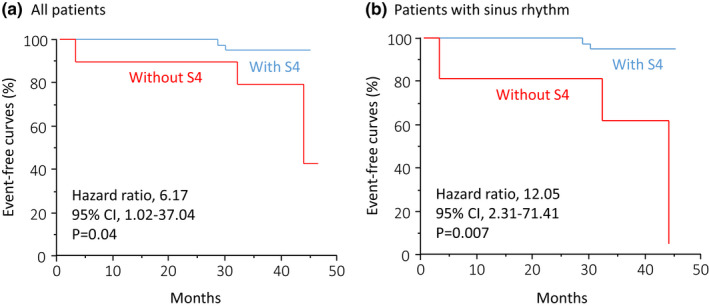
Cardiovascular outcomes. Panels A and B show Kaplan–Meier estimates of the cumulative incidence of the primary outcome in all patients and patients with sinus rhythm at the time of the CPET. CI = confidence interval; S4 = fourth heart sound.

Follow‐up data on outcomes were available for all patients. During a follow‐up period of 40.5 ± 3.2 months, six patients developed cardiac events. Among sinus rhythm patients with S4, three developed paroxysmal AF, whereas persistent AF developed in three out of five sinus rhythm patients without S4, two of whom had heart failure. No cardiac death or stroke occurred. The incidence of the primary outcome was higher in patients without S4 than in those with S4 (33% vs. 8%; hazard ratio, 6.17; 95% CI, 1.02 – 37.4; *p* = .04) and higher in sinus rhythm patients without S4 than in those with S4 (60% vs. 8%; hazard ratio, 12.05; 95% CI, 2.31 – 71.41; *p* = .007; Figure 3).

## 8 DISCUSSION

This study revealed that the incidence of S4 was high in patients with HCM and that the absence of S4, as assessed by phonocardiography, was significantly associated with exercise intolerance. Furthermore, HCM patients without S4, particularly sinus rhythm patients without S4, were more likely to develop adverse cardiac events during the follow‐up than sinus rhythm patients with S4.

A key result of this study was the relationship observed between absent S4 and exercise intolerance. The underlying mechanism remains unclear because reduced exercise tolerance is considered to be multifactorial. However, since atrial function has been shown to play a pivotal role in the exercise capacity of HCM (Briguori et al., 1999; Sachdev et al., 2005), the present results suggest that atrial function was more impaired in sinus rhythm patients without S4, as observed in patients with AF, than in those with S4. This speculation may be supported by the results of echocardiography in this study. Left atrial dimensions and mitral valve E wave peak velocities were the highest in patients with AF, middle in sinus rhythm patients without S4, and the lowest in sinus rhythm patients with S4. Similar echocardiographic findings were reported for HCM patients with persistent AF, paroxysmal AF, and no AF in another study (Azarbal et al., 2014).

A gradual decrease in atrial function may contribute to the loss of the ability to produce S4, even while still in sinus rhythm on electrocardiography. In an analysis of exercise capacity in patients with HCM who underwent symptom‐limited CPETs, not only patients with AF, but also those with paroxysmal AF showed lower peak VO_2_ than patients without AF, although patients with paroxysmal AF showed sinus rhythm at the time of CPETs (Azarbal et al., 2014). It is thus not surprising that sinus rhythm patients with impaired atrial function are more likely to develop paroxysmal AF or proceed to persistent AF. Furthermore, atrial and brain natriuretic peptide levels were higher in sinus rhythm patients without S4 than in those with S4 in this study; both natriuretic peptides are released in response to cardiac stress (Yasue et al., 1994).

Another important result of this study is that absent S4 was associated with adverse cardiac events, which was mainly driven by the development of AF. AF is responsible for the symptoms observed in patients with HCM and markedly interferes with quality of life, leading to an increased risk of death (Kirchhof et al., 2016; Olivotto et al., 2001), even in patients with apical HCM (Lee et al., 2017). Furthermore, increased risks of HCM‐related mortality and functional deterioration have been reported in patients who progressed from paroxysmal AF to chronic AF (Olivotto et al., 2001). In a retrospective analysis of more than 4000 HCM patients who previously had no documented AF, a multivariable Cox’s regression analysis revealed a relationship between AF and female sex, age, left atrial diameter, New York Heart Association class, hypertension, and vascular disease (Guttmann et al., 2017). Since AF may be an uncommon primary cause of death when treated appropriately (Rowin et al., 2017), the present results appear to be of clinical value in the management of HCM. Further research is needed to confirm whether absent S4 is an additional risk factor for AF in HCM patients with sinus rhythm.

There are some limitations that need to be discussed. This study was retrospectively conducted at a single center, and our patients were highly selected, suggesting that the results obtained cannot be extrapolated to the general HCM population. Furthermore, in this study, S4 was assessed using phonocardiography, which may be limited in availability. However, the concordance regarding S4 between phonocardiography and auscultation by an experienced cardiologist is clinically acceptable (Sato et al., 2018). In addition, S4 may wax and wane because hemodynamics dynamically changes in patients with HCM. Another limitation is that paroxysmal AF may have been underdiagnosed in our patients with HCM. This may be inevitable because paroxysmal AF may be asymptomatic and its complete detection needs an implantable device; patients with these devices were excluded from this study.

In conclusion, the absence of S4 on phonocardiography was associated with impaired exercise tolerance and adverse cardiac events, especially newly developed AF, in patients with HCM. Further research is needed to examine whether absent S4 is a reliable marker for the development of AF and adverse cardiac events in this condition.

## 9 ETHICS

The Ethics Committee of Matsushita Memorial Hospital. Referencenumber: 19036.

## 10 DATA AVAILABILITY STATEMENT

No data are available. The authors declare that data supporting the findings of this study are available within the article.

## CONFLICT OF INTEREST

None declared.

## Data Availability

The deidentified participant data will not be shared.
